# Systematic analysis of the underlying genomic architecture for transcriptional–translational coupling in prokaryotes

**DOI:** 10.1093/nargab/lqac074

**Published:** 2022-09-27

**Authors:** Richa Bharti, Daniel Siebert, Bastian Blombach, Dominik G Grimm

**Affiliations:** Technical University of Munich, Campus Straubing for Biotechnology and Sustainability, Bioinformatics, Petersgasse 18, 94315 Straubing, Germany; Weihenstephan-Triesdorf University of Applied Sciences, Petersgasse 18, 94315 Straubing, Germany; SynBiofoundry@TUM, Technical University of Munich, Schulgasse 22, 94315 Straubing, Germany; SynBiofoundry@TUM, Technical University of Munich, Schulgasse 22, 94315 Straubing, Germany; Technical University of Munich, Campus Straubing for Biotechnology and Sustainability, Microbial Biotechnology, Uferstraße 53, 94315 Straubing, Germany; SynBiofoundry@TUM, Technical University of Munich, Schulgasse 22, 94315 Straubing, Germany; Technical University of Munich, Campus Straubing for Biotechnology and Sustainability, Microbial Biotechnology, Uferstraße 53, 94315 Straubing, Germany; Technical University of Munich, Campus Straubing for Biotechnology and Sustainability, Bioinformatics, Petersgasse 18, 94315 Straubing, Germany; Weihenstephan-Triesdorf University of Applied Sciences, Petersgasse 18, 94315 Straubing, Germany; SynBiofoundry@TUM, Technical University of Munich, Schulgasse 22, 94315 Straubing, Germany; Technical University of Munich, Department of Informatics, Boltzmannstr. 3, 85748 Garching, Germany

## Abstract

Transcriptional-translational coupling is accepted to be a fundamental mechanism of gene expression in prokaryotes and therefore has been analyzed in detail. However, the underlying genomic architecture of the expression machinery has not been well investigated so far. In this study, we established a bioinformatics pipeline to systematically investigated >1800 bacterial genomes for the abundance of transcriptional and translational associated genes clustered in distinct gene cassettes. We identified three highly frequent cassettes containing transcriptional and translational genes, i.e. *rplk-nusG* (gene cassette 1; in 553 genomes), *rpoA-rplQ-rpsD-rpsK-rpsM* (gene cassette 2; in 656 genomes) and *nusA-infB* (gene cassette 3; in 877 genomes). Interestingly, each of the three cassettes harbors a gene (*nusG, rpsD* and *nusA*) encoding a protein which links transcription and translation in bacteria. The analyses suggest an enrichment of these cassettes in pathogenic bacterial phyla with >70% for cassette 3 (i.e. *Neisseria*, *Salmonella* and *Escherichia*) and >50% for cassette 1 (i.e. *Treponema*, *Prevotella*, *Leptospira* and *Fusobacterium*) and cassette 2 (i.e. *Helicobacter*, *Campylobacter*, *Treponema* and *Prevotella*). These insights form the basis to analyze the transcriptional regulatory mechanisms orchestrating transcriptional–translational coupling and might open novel avenues for future biotechnological approaches.

## INTRODUCTION

Prokaryotes lack, in contrast to eukaryotic cells, a distinct nuclear compartment which allows the spatiotemporally coupling of transcription and translation (1–3). In this process, ribosomes attach already to the still growing mRNA to start translation. Thereby, the leading ribosome follows the RNA polymerase or even physically interacts with it (4). As a result, monocistronic and polycistronic mRNAs are simultaneously formed and translated into their respective gene products (5,6). This mechanism minimizes cellular energy requirements (7) and enables high dynamics in prokaryotic gene expression. A coordinated and balanced coupling of transcription and translation seems to be essential since uncoupling may negatively affect cell viability (4,8).

The core enzyme of the bacterial RNA polymerase is composed of the five subunits α-dimer (α_2_), β, β′ and ω which form together with respective σ factor the holoenzyme (9). Bacterial ribosomes consist of the small (30S) and large (50S) subunit. The small subunit is formed of 16S rRNA and 21 ribosomal proteins (designated S1–S21) whereas the large subunit is composed of 23S and 5S rRNA and 33 ribosomal proteins (designated L1–L36) (10). The coding sequence, structure and function of these components are evolutionarily conserved from prokaryotes to eukaryotes (11–13). However, structural analysis of the ribosomal complexes is still subject of current research (14) and allows new insights also in transcriptional–translational coupling (15). NusA and NusG are the major regulators of bacterial transcription elongation and alter the properties of the transcription elongation complex (16). Recent studies suggest that NusA and NusG are also relevant for resynchronization of transcriptional–translational coupling (16,17). Moreover, by contacting both RNA polymerase and the NusE/S10 protein of the leading ribosome, NusG can physically link transcription with translation (15,18,19). Recently, also for NusA interaction between the S2/S5 protein of the ribosome and the RNA polymerase was demonstrated (20). Regulation of transcriptional-translation coupling is also governed by the stringent response mediated by (p)ppGpp. Under diverse stress conditions such as amino acid starvation this alarmone is formed by RelA, accumulates intracellularly, and impacts the transcription of many genes such as genes encoding ribosomal proteins. However, it has been shown, that (p)ppGpp is also competitively binding to the translation initiation factor 2 (encoded by infB) and thus inhibiting translation initiation (4). Direct protein-protein interaction between ribosomal proteins (e.g. S1, S4 and S11) and the RNA polymerase has also been reported (21–23). Besides their primary role as integral components of the ribosome, some ribosomal proteins additionally show extra ribosomal activity with so called moonlighting function (24) as such they exert regulatory functions and act as, e.g. repressors inhibiting translation of their own mRNAs to keep ribosomal protein homeostasis (24,25).

For several well investigated organisms, such as *Escherichia coli* or *Bacillus subtilis*, the occurrence of operons which contain besides other genes for transcription and translation are known for decades (26,27). However, a comprehensive analysis of the underlying genomic architecture over a wide range of bacterial genomes is not available so far.

As a prerequisite for proper transcriptional–translational coupling, we hypothesized in this study that (selected) genes encoding elements of the transcription and translation machinery are organized in a gene cassette. Such a genomic architecture might facilitate orchestrating gene expression, has a functional relevance to prokaryotic survival and therefore has resulted from evolutionary selection. Therefore, we systematically analyzed the gene cassettes from 1800 bacterial genomes using state-of-the-art bioinformatics, statistics, and data-mining approaches. We identified cassettes containing genes relevant for transcription and translation across prokaryotic genomes with high abundance that might function as coordinated regulatory module(s). The top three gene cassette also harbor genes which are directly associated with transcriptional–translational coupling indicating a coordinated expression to facilitate this mechanism in prokaryotes.

## MATERIALS AND METHODS

### Selection of genomes and extraction of gene cassettes

To identify and investigate gene cassettes containing both transcriptional and translational genes, 2071 genomes (consist of 1939 bacteria and 133 archaea) were downloaded from the DOOR2 (the Database of prOkaryotic OpeRons) database and the corresponding annotation files were retrieved from the NCBI Assembly database using the available REST API (28).

Knowledge about operons and the availability of complete prokaryotic genomic sequences have enabled the *in-silico* prediction of operons based on sequencing data. However, a sequence-based elucidation of operons is challenging. It has been observed in different transcriptomic studies that an operon may have different variations in its component genes expressed under different conditions, termed as transcriptional units (TUs) (29). The current version of the DOOR database consists of two types of operons: (i) operons predicted based on sequences and (ii) a limited number of TUs identified using transcriptomic data. The DOOR databases uses a discriminative machine learning model to predict operons using experimentally validated operons from a few organisms, including *Escherichia coli* and *Bacillus subtilis* (30). Based on whether the target genome has a substantial number of experimentally identified operons or not, two separate classifiers are trained. For the first case, the model was trained using a nonlinear (decision tree-based) classifier utilizing both general features of genomes and genome-specific features for a known subset of the operons. For the second case, the model was trained using a linear (logistic function-based) classifier, based only on general features of genomes (30). According to the method used in DOOR2 an operon classifies each pair of adjacent genes into two classes: *in* or *not in* the same operon, using five features:

1. **Intergenic distance**: The intergenic distance (${D_I}$) between each adjacent gene pair is calculated as ${D_I}\ = \ downstream\_gene\_start\ - \ ( {upstream\_gene\_end\ + \ 1} )$. On observing distributions of ${D_I}$ in experimentally validated *E. coli* and *B. subtilis* it was found that there are only a small number of ${D_I}$ values that are lower than –50 (i.e. two genes whose sequences are overlapped by 50 nt) and most of known gene pairs with ${D_I} >250$ are found to be boundary pairs. Therefore, the ${D_I}$ values –50 and 250 are used as the lowest and highest cutoff values, respectively (31).
2. **Conservation level of the two genes in the same neighborhood across other genomes**: Neighborhood conservation of two genes is based on a score which is calculated by the log-likelihood of the probabilities of the gene-pair in each genome. The value of the score determines if the gene-pair is present in the neighborhood or not. Smaller scores are generally associated with gene pairs that are functionally related (32).
3. **Functional relatedness**: Functional relatedness is measured using phylogenetic distances between two genes. The smaller the distance, the more functionally related the genes are. The phylogenetic distance between a pair of genes is calculated based on the Hamming distance and the Shannon entropy (32).
4. **The ratio between the lengths of the two genes**: The length ratio between a pair of genes is the score calculated as the natural logarithm of the length ratio of the upstream and downstream gene. This feature is most valuable when the training and testing data are from the same genome and is generally useful for operon prediction (32).
5. **Frequencies of certain predefined DNA motifs in their intergenic region**: DNA motifs are included in the operon prediction based on the strength between operon pairs and boundary pairs which is calculated by counting the number of occurrences for each DNA motif in the intergenic region of each gene pair. Motif frequencies are normalized values based on the count for each gene pair. The gene pair count is based on the extracted 100 nucleotides upstream of the translational start site of the downstream gene (32).

Among these features, the intergenic distance is the highest discerning feature in predicting if a pair of adjacent genes is in the same operon. After excluding genomes that are redundant and genomes lacking gene annotations or with inconsistent annotations, a total of 1974 genomes were left for further analysis.

### Identification and ranking of gene clusters

Based on the available information of genes associated with translation and transcription, the cassettes can be further divided into the following four categories:

- Genes in cassette associated with *only transcription*.
- Genes in cassette associated with *only translation*.
- Genes in cassette associated *with both transcription and translation*.
- Genes in cassette associated with *neither transcription and nor translation*.

For all 1974 genomes, gene cassette have been identified and assigned to one of these four categories. Further, a ranking across all genomes based on the number of occurrences for each of these four categories has been generated as elaborated in the section below.

### Identification of transcriptional–translational gene cassettes

The occurrence based ranking was carried out based on the number of gene cassettes, functions, and COG (‘Clusters of Orthologous Groups of proteins’) IDs across all genomes. This resulted in the identification of highly frequent genes, functions and COG IDs associated with transcription, translation, or both. The top 18 transcriptional–translational co-occurring genes were then used to perform a gene enrichment analysis for KEGG and Gene ontology (GO) terms using the R package Clusterprofiler v3.4.4 (33). We only considered pathways as significantly enriched after multiple hypothesis correction using Benjamini-Hochberg with an FDR based threshold of α ≤ 0.05. The STRING v10 (34,35) database has been used to perform a network based analysis for clustering gene cassettes based on gene fusion (genes reportedly existing as hybrids without any intergenic sequence(s)), gene neighborhood (genes within close proximity) and gene co-occurrence (genes existing together on same genomic loci with intergenic sequences and/or other genes). Finally, the frequency of the resulting gene cassettes across all extracted genomes from the DOOR2 database were computed. Here, it is important to note that due to inconsistency in the annotations, the computed numbers are only based on the extracted data from the annotations from the NCBI Assembly database and only depict a trend and not the final count.

### Data and code availability

A detailed step-by-step analysis protocol for all bacterial gene cassettes reported in this paper has been created and is freely available for download on GitHub: https://github.com/grimmlab/transcriptional-translational-coupling. The code repository includes all necessary tools, algorithms, and analysis scripts to reproduce the results and some figures from this paper.

## RESULTS

### Extraction and segregation of gene cassettes

**Figure 1.**
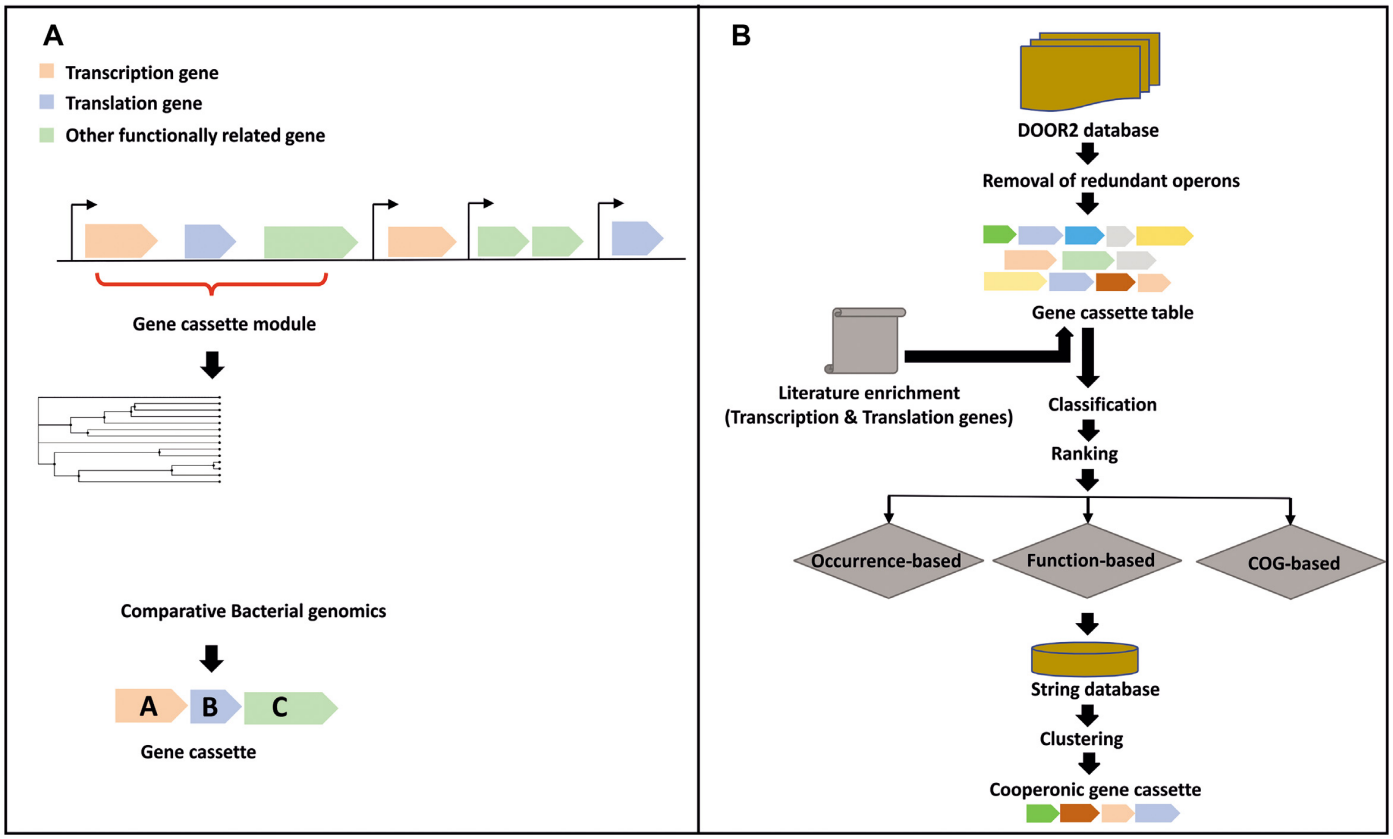
(**A**) Flowchart illustrating the pipeline for extracting gene cassette data of bacterial transcriptional and translational genes (see Materials and Methods section and analysis pipeline at GitHub). (**B**) Process map of extracting transcriptional–translational gene cassettes. Individual GenBank annotation files obtained for individual DOOR2 operons list were used as input. Following a three-way ranking based on gene-occurrence, gene-function and COGs, highly frequent transcriptional–translational genes were identified. These were analyzed using a protein-network based on the STRING v10 database which led to the identification of gene cassettes.

The present work is based on the hypothesis that several operonic modules are conserved in bacteria and operate together to coordinate coupled transcriptional–translational mechanisms. We created a comparative genomics pipeline for screening genomic distributions of these probable conserved gene cassettes in bacteria (Figure 1A). A total of 1974 genomes with locus tag information were used for the final analysis. Out of these 1974 genomes, 1843 genomes contained both, transcriptional and translational gene cassettes. All further downstream analyses have been conducted on these 1843 genomes. First, annotation files from the NCBI Assembly database have been extracted for each genome and a table of gene cassettes was created based on the relative proportions of cassettes which fall into one of the following categories: (i) transcriptional, (ii) translational, (iii) both, transcriptional & translational and (iv) none. Second, count data was generated for each gene cassette table from the previous step by comparing them with a comprehensive list of bacterial transcriptional and translational genes (Figure 1B).

The resulting gene list consists of gene names and their reported synonyms for each individual entry. A simultaneous keyword (gene name) and synonym-based (gene-synonym) search module was utilized to create a count table containing a catalogue of each of the three categories. A total of 1710 gene names (Sheet 1, Supplementary File S1), 4008 functions (Sheet 2, Supplementary File S1) and 1499 COG IDs (Sheet 3, Supplementary File S1) across 1843 bacterial genomes were compiled into a count table (Supplementary File S1). This segregated gene list was further utilized to identify highly frequent gene clusters associated with bacterial gene cassettes.

### Ranking and distribution analysis of gene clusters

Genes having similar or overlapping functions often cluster together which helps them perform coordinated regulatory roles inside the cellular milieu (36). This clustering is often conserved as it provides selection benefits for various complementary functions including transcription-translation coupling in bacteria (37,38). Thus, a three-way ranking system based on genome-wide occurrence frequency of gene names, functions and COG IDs in the count table was utilized to identify highly frequent genes present in the gene cassette modules. Next, the relative proportions of gene cassette in each category, i.e. transcription, translation, and both, together with their functional annotations were computed. Only the genes falling into *both* categories (transcription and translation) were compiled and ranked based on a frequency cut-off of 300 genomes (Supplementary File S1).

**Figure 2.**
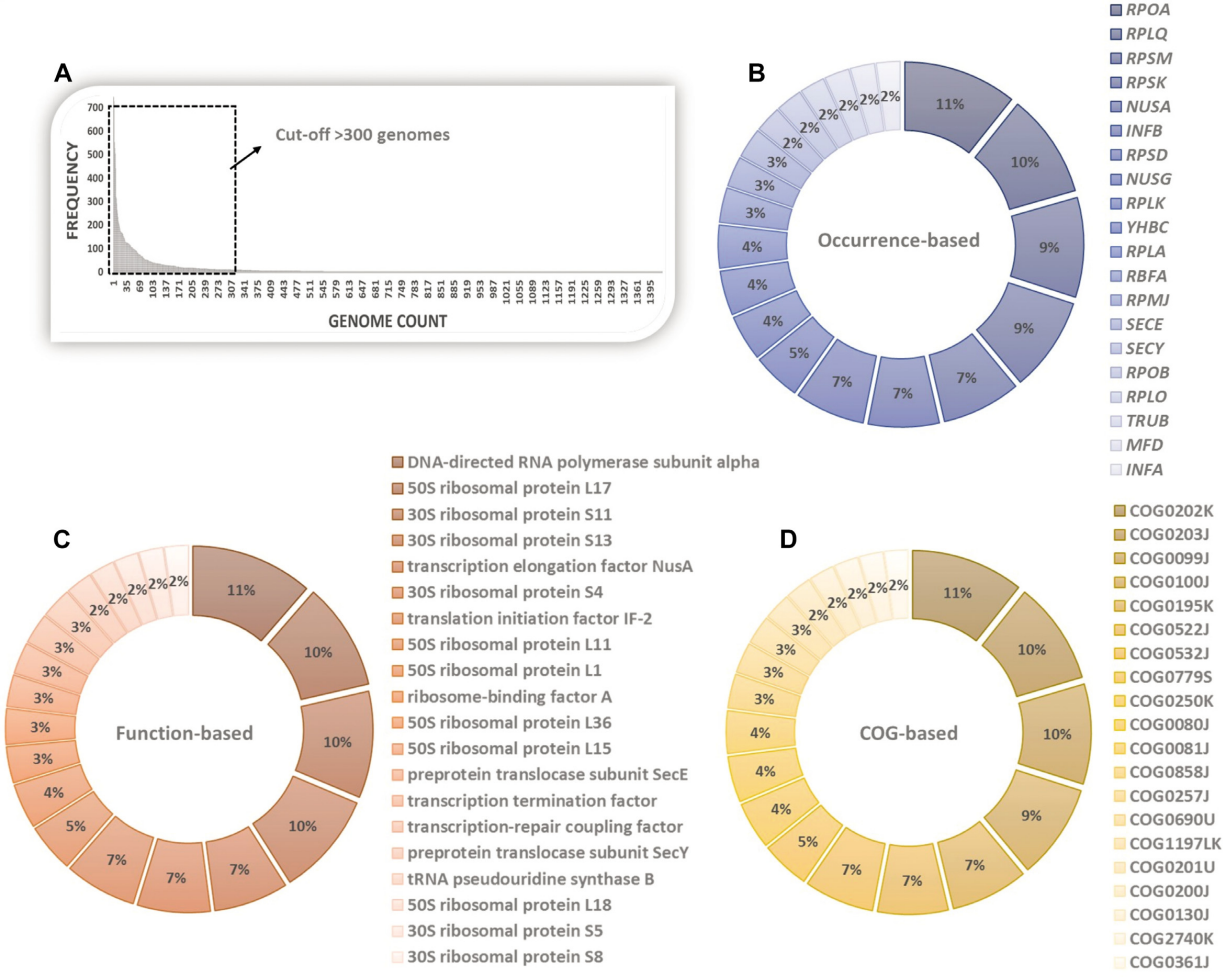
Distribution analysis of individual DOOR2 extracted bacterial cassette genes and (**A**) extraction of top 20 genes by ranking based on (**B**) Gene-occurrence distribution (top to bottom: rpoA, rplQ, rpsM, rpsK, nusA, infB, rpsD, nusG, rplK, yhbC, rplA, rbfA, rpmJ, secE, secY, rpoB, rplO, truB, mfd, infA), (**C**) Function-based (gene product/protein) distribution and (**D**) COG-based distribution. In each case, the pie-charts indicate top 20% of ranked genes, functional gene-product/proteins and COG IDs.

The gene-based ranking involved creating a genome occurrence-based frequency table of all genes (Sheet 1, Supplementary File S1). Individual genes/gene names were compiled based on their frequency of occurrences in the list of bacterial genomes. Our data showed the highest frequency of occurrence for the DNA-directed RNA polymerase subunit alpha (encoded by *rpoA*; 1442 genomes) followed by the *nusA* gene encoding a transcription elongation regulator and three other ribosomal proteins (encoded by *rplQ*, *rpsM* and *rpsK*) with occurrence in more than 1000 genomes each (Figure 2B). Interestingly, 80% of the top 20 genes are translationally associated genes and the remaining 20% are associated with the transcriptional machinery (Figure 2A, Table 1, and Supplementary File S1).

**Table 1.** Top 20 genes extracted from gene cassette library based on gene occurrence, Functional proteins, and COGs. The gene name of each entry is provided. The cut-off for frequency of genome occurrence was chosen as >300 genomes

Gene	Frequency	Function	Gene name	Frequency	COG ID	Gene name	Frequency
*rpoA*	1442	DNA-directed RNA polymerase subunit alpha	*rpoA*	1663	COG0202K	*rpoA*	1463
*rplQ*	1321	50S ribosomal protein L17	*rplQ*	1462	COG0203J	*rplQ*	1337
*rpsM*	1269	30S ribosomal protein S11	*rpsK*	1453	COG0099J	*rpsM*	1303
*rpsK*	1201	30S ribosomal protein S13	*rpsM*	1412	COG0100J	*rpsK*	1239
*nusA*	1004	transcription elongation factor NusA	*nusA*	1014	COG0195K	*nusA*	1006
*infB*	901	30S ribosomal protein S4	*rpsD*	992	COG0522J	*rpsD*	917
*rpsD*	890	translation initiation factor IF-2	*infB*	980	COG0532J	*infB*	908
*nusG*	609	50S ribosomal protein L11	*rplK*	662	COG0779S	*rimP*	630
*rplK*	579	50S ribosomal protein L1	*rplA*	610	COG0250K	*nusG*	611
*yhbc*	565	ribosome-binding factor A	*rbfA*	494	COG0080J	*rplK*	596
*rplA*	543	50S ribosomal protein L36	*rpmJ*	487	COG0081J	*rplA*	553
*rbfA*	441	50S ribosomal protein L15	*rplO*	432	COG0858J	*rbfA*	444
*rpmJ*	382	preprotein translocase subunit SecE	*secE*	430	COG0257J	*rpmJ*	395
*secE*	380	transcription termination factor	*nusG*	418	COG0690U	*secE*	372
*secY*	334	transcription-repair coupling factor	*mfd*	417	COG1197LK	*mfd*	351
*rpoB*	334	preprotein translocase subunit SecY	*secY*	356	COG0201U	*secY*	337
*rplO*	317	tRNA pseudouridine synthase B	*truB*	336	COG0200J	*rplO*	324
*truB*	316	50S ribosomal protein L18	*rplR*	331	COG0130J	*truB*	319
*mfd*	310	30S ribosomal protein S5	*rpsE*	330	COG2740K	*ylxR*	307
*infA*	302	30S ribosomal protein S8	*rpsH*	325	COG0361J	*infA*	302

Similarly, function-based frequency and COG-based ranking was performed based on functional gene distribution and frequency of COG IDs within the bacterial genome list. (Figure 2C and D). Here, the data shows the presence of crucial transcriptional genes *rpoA* and *nusA* along with 50S and 30S ribosomal fragments with high frequency (>1000 genomes) of occurrence (Table 1 and Supplementary File S1). Based on these rankings, we observed the highest frequency for genes of transcriptional components (*rpoA*, *nusA*) followed by different ribosomal subunits and other translation associated components with distributions of >1000 COG terms (Table 1 and Supplementary File S1).

### Identification of high frequency gene cassettes

**Figure 3.**
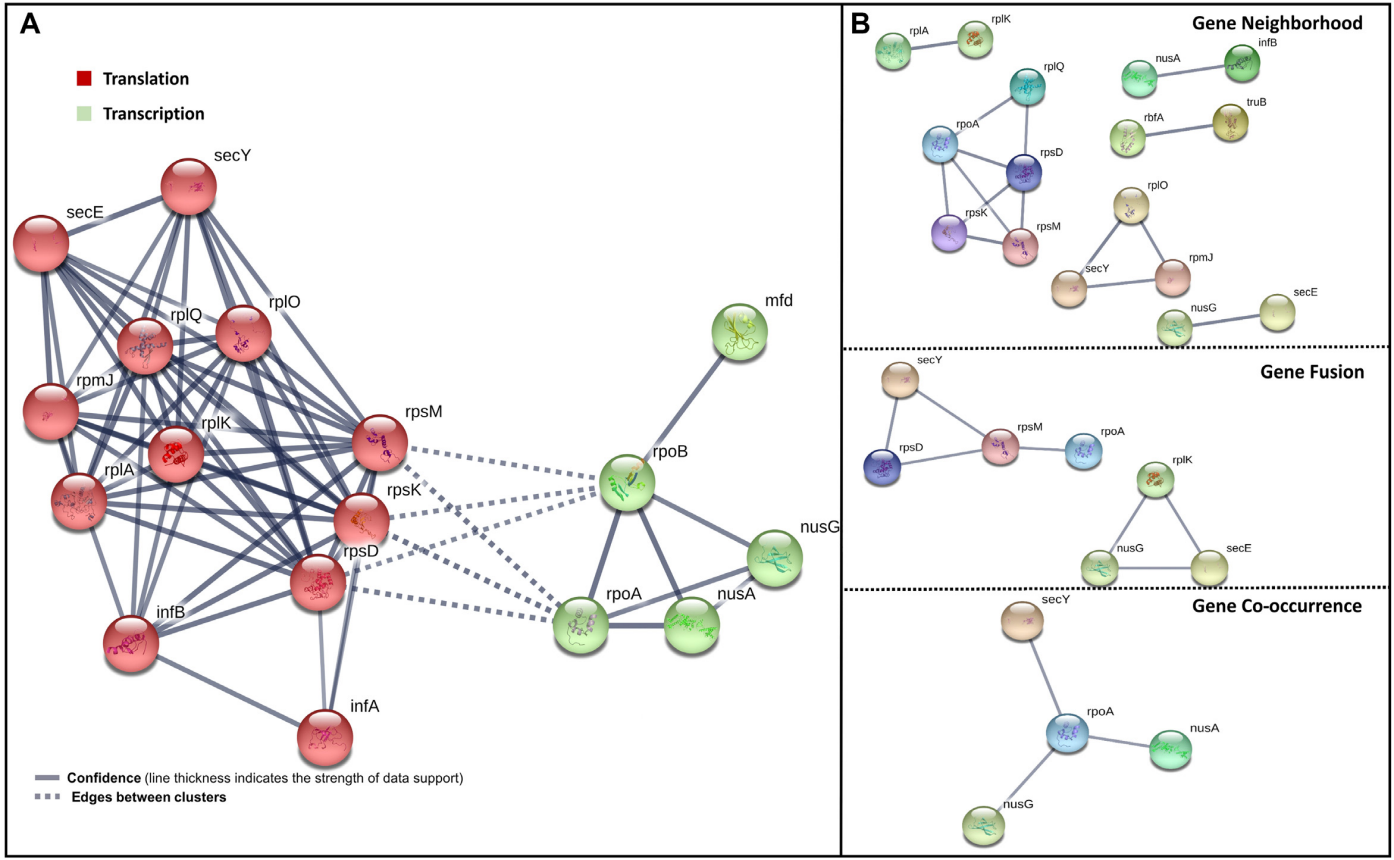
(**A**) Functional STRING Protein–Protein Interaction network with color coding is based on *k*-means clustering (*k* = 2) containing network of transcriptional and translational genes identified from top genes. (**B**) Identified distinct gene cassette based clustering on three criteria for linkage: gene fusion, gene co-occurrence and gene neighborhood. The dashed lines show the network between transcriptional and translational modules and based on experimental interaction data. For each case, a statistical cutoff of 0.7 in STRING v10 was chosen. High frequency gene cassettes were based on top 18 genes common in occurrence and GO-based distribution data.

Further, the analysis of frequency tables based on gene occurrence, function and COG-terms was done for identifying common highly frequent genes present in the genome library. Together, the frequency distribution indicated that 70% of the top 20 genes consisted of translational genes while the remaining 30% genes corresponded to the transcriptional machinery. Next, using *k*-means clustering STRING v10 Protein–Protein Interaction (PPI) networks were clustered into networks of transcriptional and translational genes that yielded in a final count of 18 highly frequent genes (34,35). These highly frequent genes were utilized for identifying highly frequent distinct gene cassettes present in the entire genome list. These cassettes could be understood as gene modules or units that might exist and coordinate together to regulate coupled transcription-translation in bacteria (Figure 3A). Further analyses were generated using settings with high confidence of 0.7 and three criteria for linkage: gene fusion, gene co-occurrence and gene neighborhood for the top identified genes (Figure 3B). Pairwise scores greater than 0.7 (from the STRING v10) were utilized to screen the identified cassettes subsequently.

With the mere identification of genes, clusters can never provide cassette information in absence of genome distribution information. Thus, the next step was to identify the frequency distribution of the identified gene clusters in our genome library. Based on the frequency distribution, three high frequency gene cassettes were identified with a frequency ranging between 300 and 700 genomes (highlighted in bold, Table 2). The *rplk-nusG* (referred as gene cassette 1) cassette could be found in 553 genomes (Sheet 1, Supplementary File S2). Further, *rpoA-rplQ-rpsD-rpsK-rpsM* (referred as gene cassette 2) was present in 656 genomes (Sheet 2, Supplementary File S2). Finally, *nusA-infB* (referred as gene cassette 3) found in 877 genomes was the most frequent cassette (Sheet 3, Supplementary File S2).

**Table 2.** Different gene cassettes identified based on three criteria for linkage i.e. gene fusion, gene neighborhood and gene co-occurrence. For each motif, the genome count and frequency are also given. The bold highlighted cassettes were chosen for further analysis based on genome frequency cut-off >300 and its molecular function

Clustering method	Motif	Genome counts	Role
**Gene fusion**	*rpsD, secY, rpsM, rpoA*	145	Transcription/Translation
	***rplK, nusG***	**553**	**Transcription/ Translation**
**Gene co-occurrence**	*secY, rpoA, rpoB,nusA, nusG*	0	Transcription/Translation
**Gene neighbourhood**	***rpoA, rplQ, rpsD, rpsK, rpsM***	**656**	**Transcription/Translation**
	*rbfA, truB*	273	Translation
	*rplA, rplk*	534	Translation
	***nusA, infB***	**877**	**Transcription/Translation**
	*secY,rplO, rpmJ*	169	Translation
	*rplO, secY*	306	Translation

It is important to note that all three cassettes were found in different operonic clusters and had several other genes distributed across each cluster. We found that gene cassette 1 (*rplk-nusG*) cooccurred with the gene coding for 50S ribosomal protein L1 (*rplA*) along with genes of an essential subunit of the protein translocation channel SecYEG, i.e. protein translocase subunit SecE in more than 50% of the genomes (Sheet 1, Supplementary File S3). Interestingly, the second cassette (*rpoA-rplQ-rpsD-rpsK-rpsM*) was also found to exist independently in more than half (∼53%) of the investigated genomes (Sheet 2, Supplementary File S3). Other than that gene cassette 2 was found to cooccur with genes coding for several ribosomal proteins, such as 60S acidic ribosomal protein P1 (*rplp1*), 50S ribosomal protein L36 (*rpmJ*) and 50S ribosomal protein L18 (*rplR*) along with several other crucial proteins including tRNA pseudouridine synthase A (encoded by *truA*) and energy-coupling factor transporter transmembrane protein BioN (encoded by *bioN*), and others. Finally, gene cassette 3 (*nusA-infB*) was found to only occur with other genes and was not found to exist alone, like gene cassette 1. We found the presence of genes coding for the ribosome maturation factor RimP with gene cassette 3 in about 65% of the investigated genomes (Sheet 3, Supplementary File S3). Besides, the cassette co-occurred with genes for several ribosomal proteins including 30S ribosome-binding factor (*rbfA*), 30S ribosomal protein S15 (*rpsO*), and ribosomal large subunit pseudouridine synthase C (*rluC*) along with certain other genes, such as tRNA pseudouridine synthase B (encoded by *truB*), riboflavin synthase (encoded by *ribC*) and genes coding for certain uncharacterized proteins.

### Functional analysis of gene clusters associated with highly frequent gene cassettes

**Figure 4.**
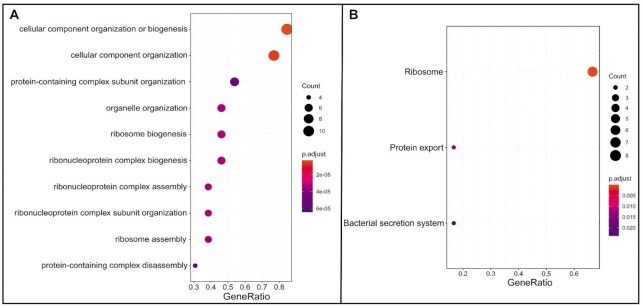
Functional enrichment analysis of coupled cassettes associated genes. (**A**) GO term based functional distribution (**B**) KEGG based functional distribution. The color bar represents the gradient of adjusted p-values and dot size represents proportion to gene ratio of the enriched gene number

Using GO and KEGG terms, the over-representation analysis was performed for gene clusters associated with the three highly frequent gene cassettes to understand their functional profiles (Figure 4). In GO based functional analyses, the most enriched term was ‘cellular component organization or biogenesis’. This corroborated with few previous publications where gene cassettes were found to be associated with energy metabolism and organelle synthesis (39–41). In fact, the preceding two functional gene clusters were associated with cellular component organization and organelle organization (Figure 4A). This is important as the other identified functional clusters were mostly associated with ribosomal assembly and organization. This indicates that due to the complexity of the ribosomal assembly, a few of the identified transcriptional machinery genes can be coupled to reduce energy consumption by limiting any excessive transcripts that cannot be translated in a timely manner in bacterial genomes (42).

The choice of this natural coupling might be based on the functional importance of the transcriptional genes that include *nusG* and *infA*. These genes are previously reported to code for bi-modal proteins that function as both negative and positive regulators of transcriptional machineries (43,44). The data was further supported by KEGG based analyses that only yielded three functional classes (Figure 4B). The top functional cluster being ribosomal assembly and associated proteins, the other two classes included protein export and secretion systems. The ‘protein export’ associated function include nuclear cytosolic export of protein to the exterior of the cell, or to the periplasmic compartment in Gram-negative bacteria (45). Finally, the bacterial secretion systems are also involved in modulating survival and nutritional mechanisms in the bacteria (46). Nevertheless, more inferences can be made only based on further analysis and experimental validations.

### The distribution of high frequency gene cassettes

After investigating the functional relevance of the three highly frequent gene cassettes their distributions were analyzed as well. All identified bacterial genomes for the three cassettes (553 genomes for gene cassette 1, 656 for gene cassette 2 and 877 for gene cassette 3, respectively) are provided in (Supplementary File S3). More than 30% of the genomes in which gene cassette 1 (*rplk-nusG*) were found belong to soil bacteria and *archaea*, including *Pseudomonas*, *Hahella*, *Halomonas*, *Pyrococcus*, *Thermococcus* and others. Interestingly, the largest cluster of genomes (>50%) in which gene cassette 1 was present belongs to pathogenic genera, such as *Treponema*, *Prevotella*, *Leptospira* and *Fusobacterium* belonging to three major bacterial phyla *Proteobacteria*, *Spirochaete* and *Firmicutes*. Similar, in gene cassette 2 (*rpoA-rplQ-rpsD-rpsK-rpsM)*, we found that the distribution was more tilted towards anaerobic bacteria and pathogens. We found bacterial genera, such as *Helicobacter*, *Campylobacter* and *Treponema* along with *Prevotella* with a distribution of more than 50%. In addition, a large cluster of soil bacteria (*Chlorobium*, *Chlorobaculum* and *Dictyoglamus)* and extremophilic archae, such as *Sulfurihydrogenibicous*, *Archaeglobus* and *Thermococcus* were also found (∼35%). Interestingly, gene cassette 3 (*nusA-infB*) showed remarkable proportions (>70%) of pathogenic bacteria genera such as *Neisseria*, *Salmonella* and *Escherichia* of the phylum proteobacteria. Here, a relatively lower abundance of soil bacteria and archaea, such as *Chlorobium, Pyrococcus* and *Thermococcus* was found.

## DISCUSSION

The present work is largely based on identifying coupled prokaryotic transcriptional and translational machineries in bacteria and archaea. This is important since the transcriptional–translational coupling in bacteria is analogous to eukaryotic nonsense-mediated mRNA decay that prevents the build-up of non-functional transcripts in the cytoplasm (47,48). We analyzed operons from the DOOR2 database to identify co-occurring and co-expressing gene clusters that are transcribed together and simultaneously translated into gene products (28).

Herein, the aim was to understand the theoretical basis of this coupling mechanism that is an important feature of gene expression in prokaryotes, where balanced and coordinated coupling is crucial for the proper function for certain bacteria cells. Previously, several gene clusters have been experimentally identified in bacteria and other prokaryotes that have a simultaneous expression mechanism (49,50). In our case, three gene cassettes that involve co-expressing transcriptional–translational genes were found with a high frequency in bacterial genomes. In these bacterial genomes a gene is considered (counted) as a gene cassette if any one of the following three conditions are satisfied: (i) if it is found in a different order; (ii) if the motif is found as a subset (all genes) within another gene cassette (again irrespective of its order); (iii) if it is found as a gene cassette itself. All three cassettes, i.e. *rplk-nusG* (gene cassette 1), *rpoA-rplQ-rpsD-rpsK-rpsM (*gene cassette 2), and *nusA-infB* (gene cassette 3) showed highly frequent distributions in >500 genomes. Interestingly, gene cassette 2 comprises the previously characterized ‘*alpha-operon*’ in bacteria. The ‘*alpha-operon*’ is a regulatory unit that comprises a set of ribosomal genes co-existing in the order of transcription *i.e. rpsM, rpsK, rpsD, rplQ* and *rpoA* (encoding RNA polymerase subunit A) (25). Importantly, the incorporated ‘*alpha-operon’* remains ‘unique’ in this gene cassette as it sits between the two ribosomal genes, i.e. *rplQ* and *rpsD* and yet get regulated independently of them (51). Interestingly, gene cassettes 1 and 3 involve genes for NusA and NusG, which remain one of the critical regulators of prokaryotic transcription elongation and can act either in concert or antagonistically (16). Both bind to RNA polymerase (RNAP), regulating pausing as well as intrinsic and Rho-dependent termination. It has been previously shown that interaction between NusA and NusG play various regulatory roles during transcription, including recruitment of NusG to RNAP, and resynchronization of transcription-translation coupling (52).

Our results indicate that transcriptional initiation might be regulated by the assembly of ribosomal proteins into the functional subunit of the translation machinery. These findings also correlate closely with a recent finding that reported direct binding of RNA polymerase with ribosomes and isolated large and small ribosomal subunits (22). It was found that RNA polymerase and ribosomes form unimolecular complexes, which get modulated by conformational and functional states of RNA polymerase and the ribosome. In fact, this direct interaction between RNA polymerase and ribosomes may constitute the three identified cassettes reported in our study that may contribute towards the transcriptional–translational coupling.

Overall, we show that the presence of three highly frequent gene cassettes may have regulatory control on the initiation of coupled transcriptional–translational mechanisms in bacteria. This is corroborated by the complexity of ribosomal assembly where a small number of transcriptional genes could be coupled to reduce energy consumption (42). The coupling phenomenon could in turn be based on the functional importance of certain regulatory transcriptional genes, such as *nusG* and *nusA*. The involvement of regulatory proteins, mainly NusG and NusA that act as dual-transcription-regulatory factors might indicate the existence of higher order operonic gene clusters in transcriptional–translational couplings in bacteria.

Additionally, the analysis showed that both gene cassettes were highly present in soil bacteria, pathogenic bacteria, and extremophiles. These sets of organisms are known to engage lower energy metabolism to survive under extreme conditions, such as high salt, and temperature, where it might be linked to their survival mechanism and robust transcriptional–translational machineries (53). Interestingly, all three cassettes showed high distributions of pathogenic bacteria with > 50% for gene cassette 1 and gene cassette 2 as well as >70% for gene cassette 3. These cassettes may act as novel targets of antisense agents that may down-regulate expression, inhibit translation and eventually terminate the pathogenic life cycle (54). Hence, our findings may pave novel ways for exploring gene cassettes that could act as new drug targets against several pathogenic bacteria. This remains a crucial avenue owing to limited efficacy of modern-day antimicrobial therapy against the emerging drug-resistant bacterial strains.

## CONCLUSION

Our study indicates the presence of highly frequent gene cassettes in bacterial genomes that might be involved in synchronizing transcription and translation. The functional enrichment analysis of gene cassette associated genes revealed enrichment of functional categories that included cellular component organization or biogenesis, and ribosome assembly along with protein export and secretion systems. On the other hand, the analysis showed the identified cassettes are highly frequent in pathogenic bacterial genera. In summary, our analysis revealed that gene cassettes might play regulatory roles in bacterial transcriptional–translational machineries and might also implicate survival benefits to certain bacterial phyla.

## DATA AVAILABILITY

A detailed step-by-step analysis protocol for all bacterial gene cassette reported in this paper has been created and is freely available for download on GitHub: https://github.com/grimmlab/transcriptional-translational-coupling. The code repository includes all necessary tools, algorithms, and analysis scripts to reproduce the results and some figures from this paper.

## Supplementary Material

lqac074_Supplemental_Files
